# A novel microchip‐based imaged CIEF‐MS system for comprehensive characterization and identification of biopharmaceutical charge variants

**DOI:** 10.1002/elps.201900325

**Published:** 2019-11-08

**Authors:** Scott Mack, Don Arnold, Greg Bogdan, Luc Bousse, Lieza Danan, Vladislav Dolnik, MaryAnn Ducusin, Eric Gwerder, Chris Herring, Morten Jensen, Jennifer Ji, Steve Lacy, Claudia Richter, Ian Walton, Erik Gentalen

**Affiliations:** ^1^ Intabio 39655 Eureka Drive Newark Newark CA USA

**Keywords:** CIEF‐MS, iCIEF, mAb, Microfluidics, Therapeutic protein

## Abstract

A microfluidic system has been designed that integrates both imaged capillary isoelectric focusing (iCIEF) separations and downstream MS detection into a single assay. Along with the construction of novel instrumentation and an innovative microfluidic chip, conversion to MS‐compatible separation reagents has also been established. Incorporation of 280 nm absorbance iCIEF‐MS analysis not only permits photometric quantitation of separated charge isoforms but also facilitates the direct monitoring of analyte focusing and mobilization in real‐time. The outcome of this effort is a device with the unique ability to allow for both the characterization and identification of protein charge and mass isoforms in under 15 min. Acquisition, quantitation, and identification of highly resolved intact mAb charge isoforms along with their critical N‐linked glycan pairs clearly demonstrate analytical utility of our innovative system. In total, 33 separate molecular features were characterized by the iCIEF‐MS system representing a dramatic increase in the ability to monitor multiple intact mAb critical quality attributes in a single comprehensive assay. Unlike previously reported CIEF‐MS results, relatively high ampholyte concentrations, of up to 4% v/v, were employed without impacting MS sensitivity, observed to be on the order of 1% composition.

**Abbreviations**
Arg: free base L arginine
BPI: base peak ion
iCIEF: imaged capillary isoelectric focusing
IDA: iminodiacetic acid

## 1 Introduction

Charge heterogeneity analysis is essential for the successful development and production of biopharmaceuticals. Since it was first reported over 30 years ago, CIEF has been proven as a highly adept approach for analyzing charge isoforms and has been widely adopted for the analysis of therapeutic proteins 1, 2, 3, 4, 5, 6. Differences in amino acid sequence, posttranslational modifications, and degradation are efficiently detected by shifts in p*I*. Unlike complementary chromatographic separations, CIEF can interrogate a protein's entire solvated structure and is less affected by changes in hydrophobicity 6. When compared to CZE, CIEF has the added advantages of a greater loading capacity and commonly supports generic platform methods that cover a wide range of p*I*.

The value of directly coupling CIEF with MS has long been recognized as a valuable analytical strategy for biopharmaceuticals. The combination of rapid, high‐resolution separations by CIEF with molecular mass identification by MS would provide a powerful and comprehensive analysis of the charge variants and their structure. Early CIEF‐MS integration involved capillary separation platforms with MS interfaces that employed coaxial sprayers 7. Though such early systems were able to analyze peptides and proteins, they had limited sensitivity and typically required manual intervention to reconfigure for the mobilization step 7, 8. Recent CIEF‐MS developments include both advancements with upstream separation methodology and ESI sprayer design. To reduce suppression and avoid the interference of nonvolatile components a 2D CIEF‐CZE separation strategy has been successfully combined to downstream MS analysis via coaxial sprayer 9. Other improvements have also led to “junction‐at‐the‐tip” CE‐MS interfaces that not only greatly reduced dilution at the ESI tip but were amenable to automated CIEF‐MS analysis 10, 11. These evolutionary designs, delivering nL/min flow rates either by hydrodynamic or electroosmotic force, have successfully performed nano‐ESI CIEF‐MS analysis of intact proteins including mAbs using commercially available CE instrumentation 12, 13, 14, 15. While impressive increases in sensitivity were achieved, all shared similar limitations, including a lack of on‐line capillary detection, manual assembly and dependency on traditional capillary‐based systems with long capillaries requiring >1 h for focusing and mobilization 16, 17.

The microchip format for integration of the CE separation to MS has been demonstrated as a promising path by several groups, including a CIEF‐MS microchip and a commercially available CZE‐MS instrument 17, 18. Microfabrication techniques can easily produce sub‐micron channel features and can offer highly reproducible, preassembled CE‐MS junction configurations, as well as provide the flexibility to reduce the effective separation length, thereby maximizing the speed and resolution of a CIEF separation. Depending on the material used for the microfluidic CIEF‐MS device, simultaneous UV transillumination, optical detection, and quantitation could also be deployed. Being able to directly observe and measure analytes during focusing and mobilization would be a highly welcomed feature, especially among analysts inside the biopharmaceutical industry where the bulk of CIEF analysis is performed. Images acquired during the CIEF‐MS separation process would greatly assist in assay development and troubleshooting as well as providing a straightforward path for comparing iCIEF‐MS results with legacy CIEF data.

Here we report on our development of a prototype integrated iCIEF‐MS system. The system features a novel iCIEF microchip that enables high‐resolution CIEF separations, 280 nm absorbance imaging of the separation channel for real‐time monitoring during focusing and mobilization, and an on‐chip ESI tip. To maximize ESI spray stability, the system monitors and maintains a constant ESI tip potential using multiple power supplies. Voltages from these power supplies are delivered by platinum electrodes through a permeable membrane barrier to avoid disrupting the CE and ESI electrical circuits with gas‐forming electrolysis products. Traditional iCIEF assays have also been converted to an MS‐compatible format. We used a trastuzumab biosimilar mAb to evaluate iCIEF‐MS system performance for intact mAb charge variant characterization. The integrated system produced high‐resolution iCIEF separations of the trastuzumab charge variants, coupled with sensitive detection and mass identification of major and minor isoforms. Five major glycoforms were detected, along with acidic and basic charge variants. Due to the system's integrated design, iCIEF‐MS analysis required only 15 min per sample.

## 2 Materials and methods

### 2.1 Equipment

CIEF separations were performed on a prototype iCIEF‐MS separation system with an incorporated CCD camera and 280 nm LED light source to perform absorbance imaging. A positive 10 kV power supply at the anode and switchable polarity 10 kV power supply at the catholyte (Cathode 1) and a negative 10 kV power supply at the mobilizer (Cathode 2) ports were used to focus the sample components and then mobilize them towards the microchip's incorporated ESI tip. Potentials from these power supplies were applied to separate platinum electrodes immersed in electrolyte tanks separated from the microchip's liquid path with MWCO 6–8 kDa membrane sheets (Spectrum Lab, PN 132677). To suppress EOF and inhibit protein adsorption, hydrophilic coated borosilicate microchips (Intabio PN MG2‐100) were used for all experimental separations. The iCIEF separation channel length as defined by the distance between the anolyte and catholyte channels was 52 mm with a depth of 40 µm and a width of 300 µm. MS detection was performed on a Bruker Compact™ QTOF with a NanoElectrospray capillary cap set installed.

### 2.2 Chemicals and reagents

All electrolyte solutions contained Optima™ LC/MS Grade water (Thermo Scientific PN W6500) and or Optima™ LC/MS Grade ACN (Fisher Chemical PN A955) solvents. Anolyte was an aqueous solution of 1% formic acid (ACROS Organics™ PN 27048). Catholyte was an aqueous solution of 1% diethylamine >99.5% Reagent Grade (Sigma Aldrich PN 47216) and the mobilizer was a 1% formic acid, 50% ACN, and 49% water.

A 500 mM cathodic spacer solution containing Free Base l‐Arginine ≥ 99.5% (Arg) (Sigma Aldrich PN 11009–100G‐F) was prepared by dissolving 0.870 mg of Arg powder into 10 mL of Optima™ LC/MS Grade water. A 200 mM anodic spacer solution containing Free Acid Iminodiacetic Acid 98% (IDA) (Sigma Aldrich PN 220000–500G) was prepared by dissolving 0.270 mg of IDA powder into 10 mL of Optima™ LC/MS Grade water. Spacer solutions were stored at room temperature.

Peptide amino acid sequences initially reported by Shimura were synthesized and individually dissolved in LC/MS grade water at 5 mg/mL to act as internal p*I* markers 19.

Prior to desalting, lyophilized mAb material was reconstituted with MS grade water. All mAb samples were desalted with a 0.5 mL Zeba™ 7K MWCO spin desalting column (Thermo Fisher Scientific PN 89882).

### 2.3 Methods

Upon mating the iCIEF instrumentation to the MS, a solution containing 100 µg/mL mAb in mobilizer was infused through the iCIEF‐MS microchip under 40–60 mbar of pressure and electrosprayed into the Bruker Compact QTOF Mass Spec equipped with a NanoElectrospray capillary cap at a potential between −3750 and −4250 volts. While monitoring the MS signal in the range from 1000–6000 *m/z*, the position of the microchip's ESI tip and MS source was optimized using a micro adjust xyz stage. To reduce the introduction of neutral species into the MS, coaxial Y and Z offset of 1 mm was set between the ESI Tip and nanospray source cone inlet. After optimizing the ESI tip position, the ESI potential was set to 200 volts.

iCIEF‐MS samples containing 250 µg/mL trastuzumab biosimilar (human, antiErbB2 antibody, research use, BioRad MCA6092), 5 mM IDA, 20 mM Arg, 1% Pharmalyte 5 to 8 Narrow Range Ampholyte, 3% Pharmalyte 8 to 10.5 Narrow Range Ampholyte, and 12.5 µg/mL peptide p*I* markers were vortexed and then degassed by centrifugation at 3900 rcf.

After priming all reagents through the microchip to the ESI tip, 5 to 10 µL of sample was flushed through separation channel. Following the introduction of sample, a short rinse of catholyte was performed to limit the sample load to the 1.3 µL volume of the separation channel spanning anolyte and catholyte channels.

After sample loading, a dark image followed by a UV transilluminated blank image were acquired by the CCD camera set in full vertical bin mode and a 10 ms exposure time. During focusing and mobilization, an image was acquired every 15 sec. These images were compared to the initial blank image to create an absorbance image. The focusing step was initiated by applying a positive 1500 V from the Anode (Anolyte) power supply while the power supply at Cathode 1 (Catholyte) was set to 0 V. Following 1 min of focusing time the anode potential was increased to 3000 V, then 2 min later the potential was further increased to 4500 V. Once 7 min of total focusing time had elapsed, a final absorbance image comprised of an average of 16 images was acquired, concluding the focusing step. To commence the mobilization step, the iCIEF‐MS system power supplies were reconfigured to apply positive Anode potential and negative Cathode 2 (Mobilizer) potential to maintain a 2300 V difference. The power supply at Cathode 1 was used to measure junction and ESI tip voltages, allowing software to adjust the Anode and Cathode 2 potentials to maintain a constant +150 V at the junction and tip. To support the Taylor Cone at the ESI Tip, the mobilizer valve was opened, and allowed to flow with 60 mbar of pressure. The Bruker Compact QTOF was also triggered at the start of the mobilization step to acquire data, scanning from 1000 to 6000 *m/z* with a 2‐scan rolling average at a 1 Hz scan rate. MS capillary potential was set between −3750 to −4250 V with drying gas set at 6 L/min and drying temperature set at 220°C.

UV traces of the focused trastuzumab biosimilar charge variants were analyzed by Protein Metrics Byos® software. Each peak in the charge profile was integrated to determine both peak area and percent composition. Mass spectra were analyzed with Bruker Compass DataAnalysis 4.4 software. Intact masses were calculated from the raw mass spectrum utilizing Maximum Entropy deconvolution with a mass range setting between 145 000 and 155 000 Da. Data point spacing was set to 3 *m/z* with normal resolution and resolving power of 8000.

## 3 Results

### 3.1 Development of the iCIEF‐MS microchip format

Transferring the iCIEF separation process into an iCIEF‐MS microfluidic chip format required the implementation of multiple novel solutions. Early experimentation revealed that high electrical resistances within the electrophoretic circuit impacted iCIEF resolution. These results guided both the channel geometry of the iCIEF‐MS chip and liquid path components. In addition to electric field‐based considerations, control of hydrodynamic flow and electrolysis gas products needed to be addressed. Completely isolating the iCIEF separation and ESI environments from unintended flow and bubbles required the application of zero dead‐volume valving and an innovative electrode design. Unlike other junction‐based CE‐MS sprayers, all high voltage or ground potentials on the iCIEF‐MS system are applied off‐chip in separate electrical assemblies. These assemblies utilize ion permeable barriers to separate an electrode tank from the liquid path to the iCIEF‐MS chip. The barriers provide not only the hydrostatic resistance needed to maintain hydrodynamic control but also prevent bubbles produced at the electrodes from entering the liquid path.

The inverse linear relationship between field strength and focused band width has been previously demonstrated in a microfluidic IEF device 20. Validating these results, poor resolution, especially in the basic end of the pH gradient, was observed in early iCIEF chip and electrode assembly designs due to their highly resistive electrical paths. Computational analysis of an early channel design predicted that only a small portion of the overall field strength was being applied to the separation channel at the start of focusing. Ultimately the chip's channel architecture was redesigned, reducing the length and increasing the width of the Anolyte, Catholyte, and Mobilizer channels, decreasing the loss of field strength. Other sources of resistance included the fluidic lines linking the electrode assemblies to the iCIEF‐MS chip and the permeable barrier material used within the electrode assemblies. Systematic reduction of electrical resistance throughout the microfluidic system dramatically increased the rate of focusing, providing peak resolution comparable to conventional iCIEF.

**Figure 1:**
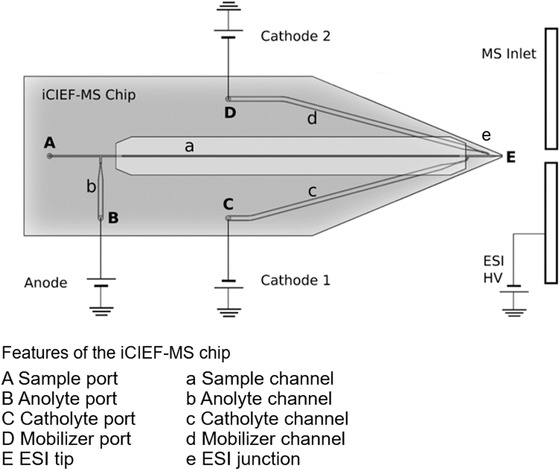
The diagram shows both the iCIEF‐MS microchip and multiple power supply high voltage system. Channels **a** through **e** were etched into a borosilicate slide and then bonded to a complementary slide with port holes **A** to **D** drilled into it. The outlet of the ESI tip **E** was generated when the two microchip slides were bonded together sealing the ESI junction channel **e**. During focusing Cathode 1 acts as a ground and electrical potential is applied by the anode to the separation channel formed between the intersection of **ab** and **ac**. Once focusing is complete, mobilization is initiated by reconfiguring the iCIEF‐MS system and triggering the MS for acquisition. ESI spray and iCIEF mobilization are simultaneously commenced by opening the valve linking a pressurized mobilizer reservoir to port **D** and applying an electrical potential between Anode 1 and Cathode 2 extending the separation channel from **ab** to **ad**. During mobilization Cathode 1 monitors ESI tip potential at intersection **ac**.

**Figure 2:**
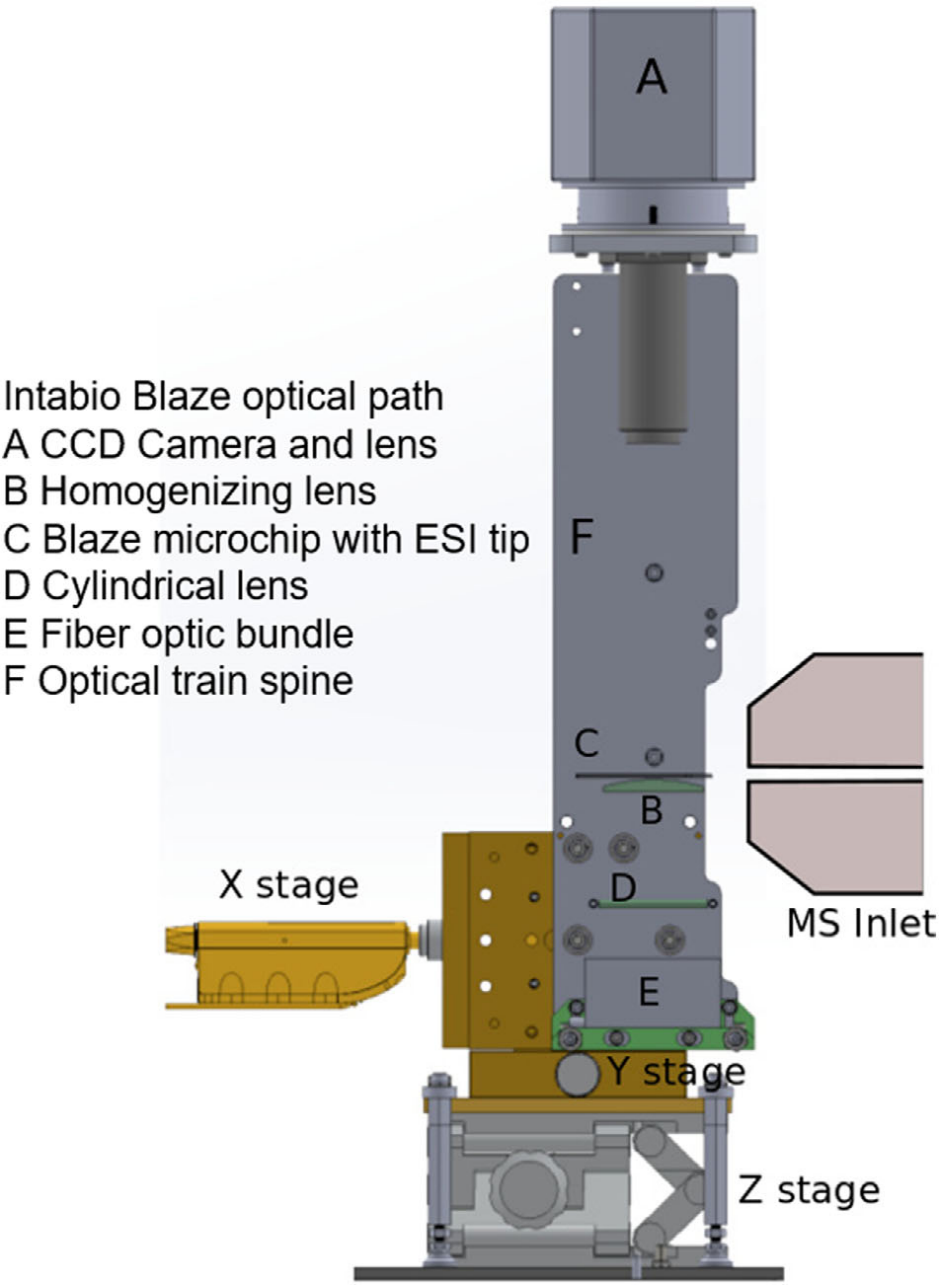
An illustration of the major iCIEF‐MS imaging system elements. The optical train **A** to **E** component alignment is maintained by being fixtured to a ridged spine **H**. The adjustability needed to align the iCIEF‐MS microchip's ESI Tip **C** to the MS Inlet is provided by a translatable XYZ Stage **G** mounted to the base of the optical spine **H**. 280 nm light generated by an LED **F** is guided via fiber‐optic bundle **E** and distributed across the imaging region of separation channel by a cylindrical **D** and homogenizing lenses **B**. Light transmitted through the imaging section of the separation channel is then focused and detected by CCD camera **A**.

Whole capillary imaging is a standard method for detecting the analyte bands of IEF, eliminating the need to transport these bands across a fixed detection window. For iCIEF‐MS we additionally incorporated peak mobilization and ESI functionality onto the microfluidic chip. Discrete on‐chip conversion between focusing and mobilization is facilitated with a mobilizer intersection (Fig. 1ad) offset from the catholyte intersection (Fig. 1ac). This staggered arrangement ensures that diffusion of anions from the mobilizer channel (Fig. 1d) into the separation channel (Fig. 1a) does not impact focusing. Analyte zones concentrated during focusing and then displaced towards the ESI tip by chemical mobilization are detected by an imaging system diagrammed in Fig. 2. The optical system is mounted on a translatable XYZ stage to allow for positional adjustment between the iCIEF Chip ESI tip and MS Inlet. To prevent ambient light from affecting UV measurements the instrument was encased in an opaque shell with a light tight door for access and sealing bracketry for mating with Bruker Compact QTOF. The last critical element in the imaging system is the iCIEF‐MS chip material, because transmission of light at 280 nm is required. Borosilicate glass was selected for the iCIEF‐MS chip manufacturing process. Absorbance measurements performed in borosilicate iCIEF‐MS chips with channels down to a 30 µm depth were made while maintaining a linear relationship between signal and path length.

### 3.2 Chemical mobilization

**Figure 3:**
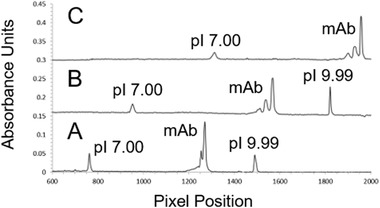
Trastuzumab biosimilar (250 ug/mL) in 1.5% Pharmalyte 5 to 8 carrier ampholyte, 1.5% Pharmalyte 8 to 10.5 carrier ampholyte, 20 mM arginine, 5 mM IDA were loaded onto an iCIEF microchip for iCIEF analysis. (A) At 6 min, focusing is complete. (B) At 8.5 min, mobilization has been initiated and peaks are electrophoretically progressing towards the ESI tip (to the right). (C) At 9.5 min, trastuzumab peaks are about to enter the ESI tip. Note the nearly baseline resolution improvement achieved between the two main trastuzumab peaks as compared to the partial resolution at 6 min when focusing is complete.

Once the focusing step of the iCIEF‐MS has been completed, the focused analyte bands must be mobilized from their positions in the separation channel to the ESI tip. Transition between the focusing and mobilization steps on the iCIEF‐MS system occurs with the near instantaneous switching of high voltage cathode terminals through software. Real‐time imaging of the chemical mobilization process ensures accurate assignment of the mass spectra to the correct CIEF peaks. The unique ability to monitor the chemical mobilization process has yielded insights into previously unobserved electrokinetic phenomena. Figure 3 contains electropherograms of a trastuzumab biosimilar mAb captured at the end of focusing and at different time points during the mobilization phase of our iCIEF‐MS assay. At the six‐minute mark, the focusing step is complete and the electropherogram image shows that the flanking peptide pI markers and mAb have focused at their respective p*I* and the mAb charge profile contains three partially resolved peaks (Fig 3A). At 8.5 min, the electropherogram shows that mobilization has been initiated and the peaks are progressing toward the ESI tip (Fig. 3B). The mAb and p*I* 7.00 and 9.99 markers have been displaced cathodically toward the ESI tip. The slope of the pH gradient decreases from to 4.1 × 10^−2^ to 3.4 × 10^−2 ^pH/pixel during the mobilization, increasing resolution between mAb charge isoforms. As mobilization continues and the mAb charge isoforms begin to migrate out of the imaged portion of the separation channel (Fig. 3C) the increased resolution between mAb charge isoform peaks is maintained. The improvement in resolution during mobilization benefits assay performance by increasing the spatial separation of the isoforms before introduction to the MS.

### 3.3 iCIEF‐MS integration

Development of an MS‐compatible iCIEF assay required the substitution of traditional CIEF reagents with volatile counterparts and the elimination of polymers from sample solutions. For example, we have successfully utilized formamide rather than urea to prevent aggregation of the focused proteins. One of the more significant challenges of coupling MS to CIEF is the ESI interface. The ESI interface must provide a stable ground for the CIEF circuit while simultaneously supporting the ESI process. Our first attempts to integrate the CIEF and ESI circuits resulted in erratic spray and MS signals. The primary cause of this instability was found to be a voltage divider effect that generated a potential at the ESI junction and tip during the chemical mobilization step. During chemical mobilization the resistance in the separation channel decreases dramatically, increasing ESI tip voltage. Grounding the CE circuit at the ESI junction would have been the most direct solution, but this would require precisely inserting an electrode into the chip and could lead to the introduction of bubbles into the Taylor Cone. Rather than altering the chip and electrode assemblies, a multiple power supply and software control system that actively cancels the voltage divider effect was developed. The system employs three power supplies, one positive polarity at the anolyte anode, one negative polarity at the mobilizer cathode and a switchable polarity power supply at the catholyte cathode. During mobilization, the current at the catholyte power supply is set to 0 µA, allowing the potential at the ESI junction to be measured through current control. The system is designed to monitor the resulting voltage and automatically adjust the positive potential at the anolyte and the negative potential at the mobilizer to maintain both the 2300 V mobilization potential difference across the separation channel and the 150 V fixed potential at the ESI tip. Incorporation of this power supply arrangement into the iCIEF‐MS instrument has permitted robust and stable ESI performance across mobilization currents from 1 to 60 µA and mobilization potentials ranging from 1 to 6 kV.

### 3.4 iCIEF MS analysis of trastuzumab biosimilar

**Figure 4:**
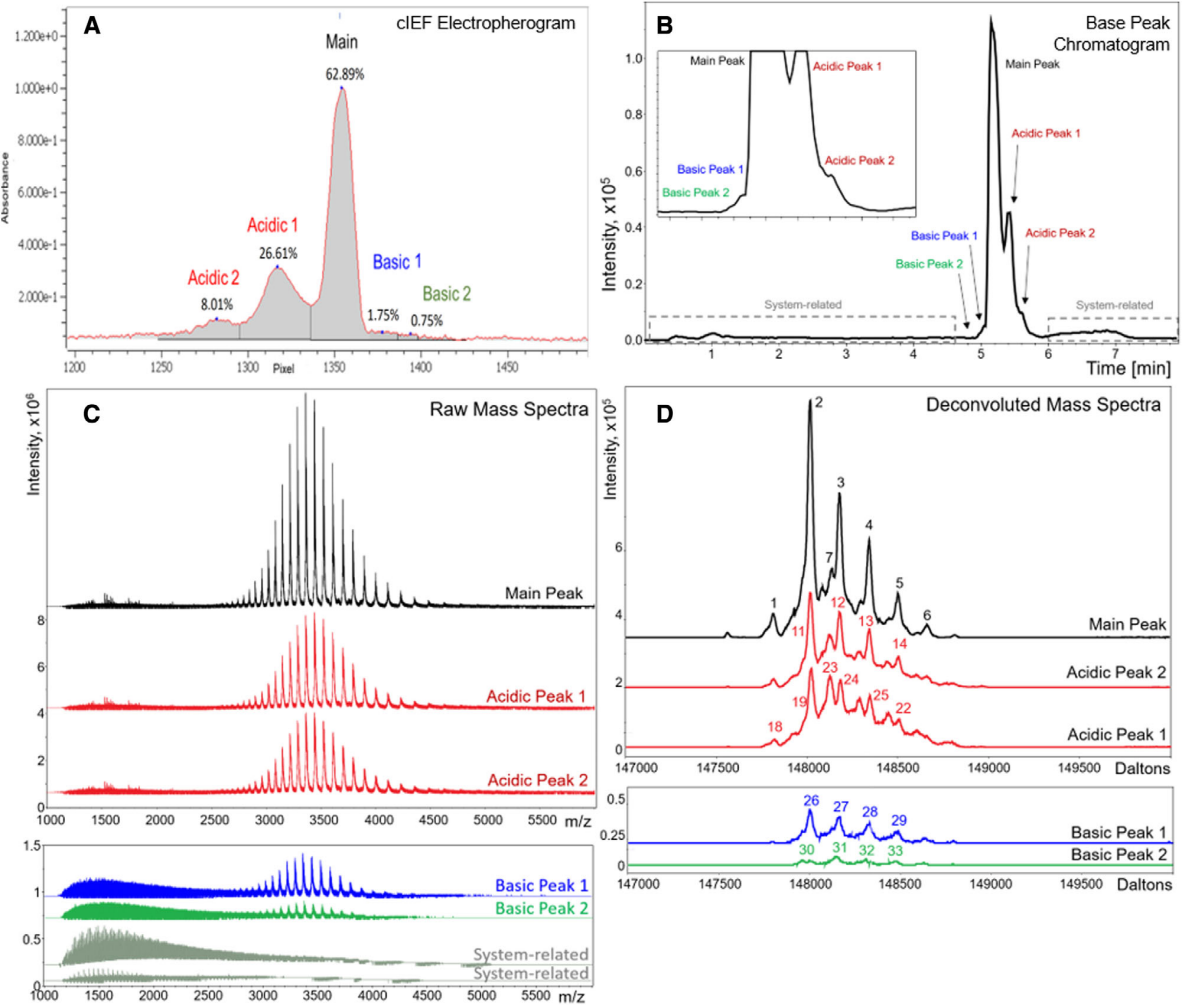
Trastuzumab biosimilar analyzed by iCIEF‐MS on a Blaze microchip. After desalting, trastuzumab was run at 250 µg/mL with 20 mM arginine, 1% Pharmalyte 5 to 8, 3% Pharmalyte 8 to 10.5, 2.5 mM iminodiacetic acid, and 10 µg/mL of p*I* markers 8.40 and 9.99. (A) Trastuzumab iCIEF UV imaged charge profile. (B) Base peak electropherogram 3000–6000 *m/z* of the mobilized trastuzumab. (C) Raw mass spectra for basic main and acidic charge variants. (D) Overlaid deconvoluted mass spectra shown for each of the charge variants.

The iCIEF‐MS system described above produces intact mAb charge envelope signals when coupled directly to a QTOF mass spectrometer. Figure 4A shows the iCIEF UV image of the trastuzumab biosimilar charge profile containing five charge isoform peaks ranging from 0.75 to 62.89% composition. Figure 4B shows the MS base peak ion trace of these same five charge variant peaks mobilized and analyzed by the QTOF mass spectrometer. It is important to note that the peaks in the MS base peak ion trace (Fig. 4B) are in the reverse order to their corresponding peaks in the iCIEF image (Fig. 4A), as basic peaks are the first to be introduced into the MS by cathodic mobilization. Maintenance of charge isoform resolution (Fig. 4A) upon introduction into the MS allows each of the five peaks to be assigned an independent time segment between the 4.9 to 5.5 min interval in the MS base peak ion trace (Fig. 4B). The raw mass spectra from each time segment show good peak symmetry from 2500 to 5000 *m/z*, indicating low interaction between the mAb ions and the iCIEF reagents (Fig. 4C). In addition, the iCIEF‐MS background spectra at <2500 *m/z* are well separated from the intact mAb charge envelopes (Fig. 4C).

Figure 4D contains the Maximum Entropy deconvoluted intact mass profiles generated from the raw mass spectra produced by each iCIEF charge isoform peak. Calculated intact mass and differences between mass isoforms from these profiles were used for identification and approximation of relative abundance (Table 1). The theoretical average mass for the most abundant isoform for the trastuzumab iCIEF main peak was predicted to be 148 022 Da. This estimation is based on the known amino acid sequence and assuming the following post translational modification: (1) all 32 cysteine residues are oxidized to form disulfide linkages, (2) the lysine residues in the C‐terminus of both heavy chains are cleaved, (3) G0F is the most abundant glycan species bound to Asn297 for both heavy chains, (4) the glutamic acid residues in the N‐terminus of both heavy chains have cyclized to form pyro‐glutamic acid. The experimental average mass for this mass variant was 148 018 Da, representing a mass accuracy of 27 ppm. The iCIEF Main Peak also included a mass variant, identified as a possible cysteinylation event due to the 120 Da increase in molecular weight. Basic Peak 1 in the iCIEF charge profile had estimated intact masses shifted by −18 Da, potentially resulting from the formation of succinimide intermediate at an Asn or Asp residue. A 128 Da increase in the measured average masses, consistent with an unprocessed Lys residue at the heavy chain C‐terminus, was observed in Basic Peak 2. A 2 Da and 1 Da mass increase in the deconvoluted mass profiles was detected with Acidic Peaks 1 and 2, respectively, indicating a deamidation. A series of overlapping unknown mass variants was also observed in both Acidic Peaks 1 and 2, which are heavier by 104 Da. Since the glycosylation pattern of these unknown acidic mAb species is comparable to the Main Peak, it is highly likely that these minor mAb species are not artifacts of the separation (Fig. 4B, Table 1). This abundant acidic modification is not typically observed in trastuzumab innovator drug, Herceptin, and could be unique to this trastuzumab biosimilar.

**Table 1:** Proposed identities of glycosylated trastuzumab main peak and minor charge variants

Peak label	Peak number	Observed average mass (Da)	Mass differences (Da)	Proposed glycan moiety
Main peak				
Main species	1	147 814	−204	G0F/G0F‐GlcNAc
	2	148 018	Main Species	G0F/G0F
	3	148 180	162	G0F/G1F
	4	148 343	325	G1F/G1F or G0F/G2F
	5	148 502	484	G1F/G2F
	6	148 662	664	G2F/G2F
Cysteinylation	7	148 138	120	G0F/G0F
	8	148 298	160	G0F/G1F
Glycosylation	9	146 572	−1446	‐/G0F
Acidic Peak 2				
Deamidation	10	147 816	−203	G0F/G0F‐GlcNAc
	11	148 019	1	G0F/G0F
	12	148 181	162	G0F/G1F
	13	148 344	326	G1F/G1F or G0F/G2F
	14	148 505	487	G1F/G2F
Unknown mAb species	15	148 122	104	G0F/G0F
	16	148 289	271	G0F/G1F
	17	148 443	425	G1F/G1F or G0F/G2F
Acidic peak 1				
Deamidation	18	147 817	−203	G0F/G0F‐GlcNAc
	19	148 020	2	G0F/G0F
	20	148 181	163	G0F/G1F
	21	148 343	325	G1F/G1F or G0F/G2F
	22	148 505	487	G1F/G2F
Unknown mAb species	23	148 122	104	G0F/G0F
	24	148 283	265	G0F/G1F
	25	148 445	427	G1F/G1F or G0F/G2F
Basic peak 1				
Succinimide intermediate	26	148 000	−18	G0F/G0F
	27	148 162	144	G1F/G1F
	28	148 324	306	G1F/G1F or G2F/G0F
	29	148 485	467	G2F/G1F
Basic peak 2				
	30	147 959	−59	Unknown
One intact C terminal lysine	31	148 146	128	G0F/G0F
	32	148 307	289	G1F/G1F
	33	148 469	451	G1F/G1F or G2F/G0F

Trastuzumab biosimilar mass isoforms grouped by iCIEF charge variant peak with observed average intact mass and mass difference from the main species peak. Proposed identity and attached glycan moieties are also listed.

## 4 Discussion

In this report, we have described key technical aspects of the design, development and application of an innovative microchip‐based iCIEF‐MS system that enables analysis of multiple critical quality attributes of an intact monoclonal antibody in a single 15‐minute assay. UV absorbance imaging of the entire separation channel enables detailed observation of the focusing and chemical mobilization phases of the assay. The imaged sample stacking and the improved resolution during mobilization enhance both the sensitivity and fidelity of the MS analysis, and suggest the first direct observation of the isotachophoretic nature of mobilization proposed by Thormann 21. Real‐time imaging also allows tracking of each peak to ensure accurate molecular mass assignment for each CIEF peak. Highly resolved charge variant separation was demonstrated for a trastuzumab biosimilar with UV detection sensitivity of <1% of the main peak. ESI of the mobilized separation components allowed for detection of mAb mass variants to a LOD below 1% composition, even with a relatively high concentration of carrier ampholytes (4% v/v). Differences between deconvoluted intact masses produced from the raw mass spectra were used to assign putative identifications for multiple charge variants and N‐linked glycan pairs. In total, 33 separate molecular features were characterized by the iCIEF‐MS system, representing a dramatic increase in the ability to monitor multiple intact mAb critical quality attributes in a single comprehensive assay. This iCIEF‐MS approach could provide substantial time savings relative to labor‐intensive offline SCX fractionation or complex 2D‐SCX‐RPLC‐MS methodologies, both of which typically require weeks to generate similar quantitation and characterization information. In addition, by coupling MS as an orthogonal mass‐sensitive second dimension to iCIEF, the integrated assay can resolve certain biologically active isoforms such as the neutral glycan structures or uncharged cystine‐linked drug payloads that would have been difficult to resolve by CIEF alone. Furthermore, the iCIEF‐MS workflow could prove highly useful in the biopharmaceutical industry where charge variant analysis and peak identification are widely utilized for various research and development activities: mAb drug candidate screening, clone selection, purification development, formulation development, stability studies, QC release testing, and various Investigational New Drug (IND)‐enabling studies.

6

*Other than the support from Intabio, Inc., the authors have declared no conflict of interest*.
